# Spermidine and spermine delay brain aging by inducing autophagy in SAMP8 mice

**DOI:** 10.18632/aging.103035

**Published:** 2020-04-08

**Authors:** Ting-Ting Xu, Han Li, Zhao Dai, George K. Lau, Ben-Yue Li, Wen-Li Zhu, Xiao-Qi Liu, Hao-Fei Liu, Wei-Wu Cai, Shui-Qing Huang, Qi Wang, Shi-Jie Zhang

**Affiliations:** 1 Science and Technology Innovation Center, Guangzhou University of Chinese Medicine, Guangzhou, China; 2 Institute of Clinical Pharmacology, Guangzhou University of Chinese Medicine, Guangzhou, China; 3 Department of Neurology, The Second Affiliated Hospital of Guangzhou University of Chinese Medicine, Guangzhou, China; 4 Touro College of Osteopathic Medicine, New York, NY 10027, USA

**Keywords:** polyamine, aging, mitochondrial dysfunction, autophagy

## Abstract

The natural polyamine spermidine and spermine have been reported to ameliorate aging and aging-induced dementia. However, the mechanism is still confused. An aging model, the senescence accelerated mouse-8 (SAMP8), was used in this study. Novel object recognition and the open field test results showed that oral administration of spermidine, spermine and rapamycin increased discrimination index, modified number, inner squares distance and times. Spermidine and spermine increased the activity of SOD, and decreased the level of MDA in the aging brain. Spermidine and spermine phosphorylate AMPK and regulate autophagy proteins (LC3, Beclin 1 and p62). Spermidine and spermine balanced mitochondrial and maintain energy for neuron, with the regulation of MFN1, MFN2, DRP1, COX IV and ATP. In addition, western blot results (Bcl-2, Bax and Caspase-3, NLRP3, IL-18, IL-1β) showed that spermidine and spermine prevented apoptosis and inflammation, and elevate the expression of neurotrophic factors, including NGF, PSD95and PSD93 and BDNF in neurons of SAMP8 mice. These results indicated that the effect of spermidine and spermine on anti-aging is related with improving autophagy and mitochondrial function.

## INTRODUCTION

Aging, the greatest risk factor for life-threatening disorders, lead to cancer, diabetes, cardiovascular and neurodegenerative conditions [1, 2]. Brain aging, a high-risk factor for dementia, changes the structure and function of brain and stimulates a gradual but detectable cognitive decline [3, 4]. The hallmarks contribute to the aging process including loss of proteostasis, dysregulated nutrient sensing, mitochondrial dysfunction and cellular senescence [5]. It is reported that the number of people aged 65 years and older will increase to 19.3% in 2030 [6], and about 1 % people suffering from dementia at the age of 60 [7]. These problems push us to probe the process, reasons and hallmarks of aging and explore the methods of anti-aging.

**Figure 1:**
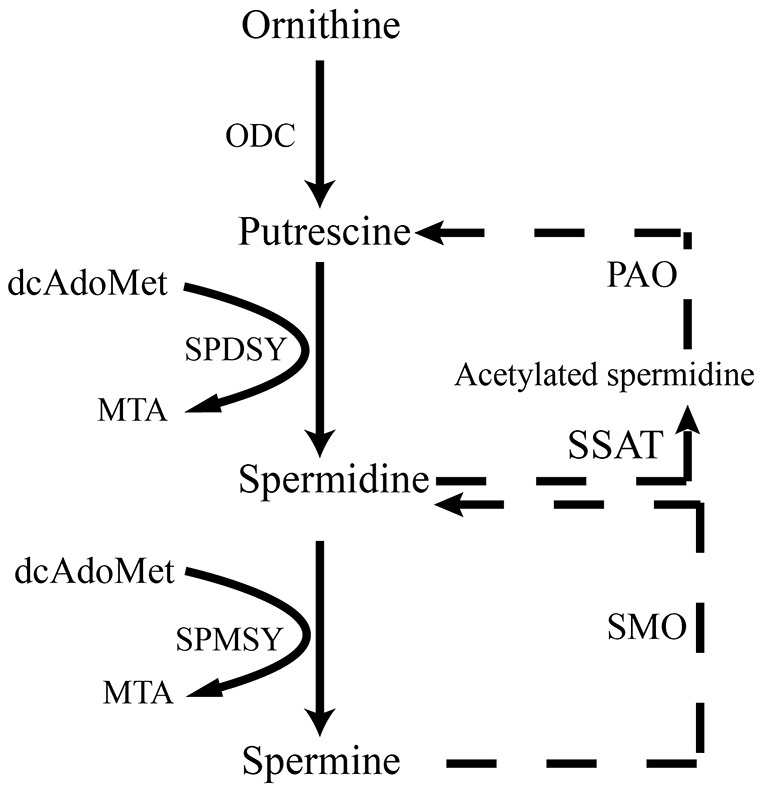
**Scheme illustrates de novo synthesis of polyamines from ornithine.** ODC, ornithine decarboxylase; dcAdoMet, decarboxylated s-adenosyl-L-methionine; SPDSY, spermidine synthase; SPMSY, spermine synthase; MTA PAO, polyamine oxidase; SSAT, spermidine/spermine N(1) acetyltransferase; SMO, spermine oxidase.

Polyamines (Figure 1), spermidine and spermine, are essential for cell viability, proliferation, function and differentiation [8–11]. The levels of polyamines in the brain of aging are gradually decrease with the progress of aging [12]. Autophagy, a self-degradative process, induces lysosomes to degrad intracellular component such as long-lived proteins, pathogen and scavenging damaged organelles [13, 14]. It is reported that autophagy plays an important role in protecting neurodegenerative disease and anti-aging [15]. The administration of spermidine and spermine induces autophagy to prolong the life of *Drosophila* and ameliorates age-induced memory impairment [16–18]. Mitochondria, the main and essential organelles, are responsible for the generation of ATP, the function and survival of neurons, the regulation of apoptosis and inflammation and reactive oxygen species (ROS) in eukaryotic cells [19–24]. Spermidine extends the lifespan of mice and exerts cardioprotective effects by enhancing cardiac autophagy, mitophagy and mitochondrial respiration [25]. Many lines of evidence support autophagy defection and mitochondrial dysfunction in the pathogenesis of aging and dementia [1, 26–31].

Based on these results, the senescence accelerated mouse-8 (SAMP8), a classical accelerated aging animal model, exhibiting systematic aging syndromes at the age of seven-month [32, 33], was employed in this study. Spermidine and spermine might ameliorate cognitive dysfunction by inducing autophagy and ameliorating mitochondrial dysfunction in SAMP8 mice.

## RESULTS

### Spermidine and spermine ameliorate memory retention loss in SAMP8 mice

**Figure 2:**
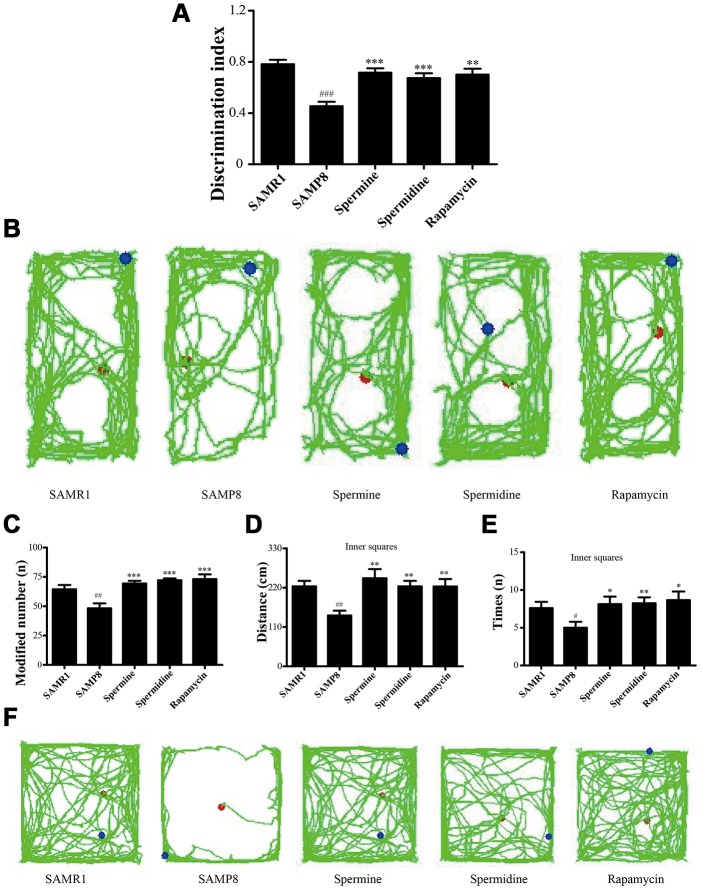
**Spermidine and spermine ameliorates cognitive dysfunction in behavioral test in SAMP8.** (**A**, **B**) Discrimination index and paths in novel object recognition. (**C**) The modified number in open field test. (**D**) The distance of inner squares in open field test. (**E**) Time spent in the inner squares in open field test. (**F**) The paths of respective groups. Data represent mean ± SEM (*n* = 10 per group). ^#^*P* < 0.05, ^##^*P* < 0.01, ^###^*P* < 0.001 vs. SAMR1; *P < 0.05, **P < 0.01, ***P < 0.001 vs. SAMP8.

Novel object recognition test (ORT) and open field test (OFT) were used to investigate the neuroprotective effect of spermidine and spermine on SAMP8 mice. After the treatment of spermidine, spermine and rapamycin, the ability of memory retention was greatly improved (Figure 2) in SAMP8 mice. Spermidine, spermine and rapamycin significantly improved the time duration of exploring the novel object (Figure 2A, 2B) in ORT. Mice in spermidine, spermine and rapamycin groups were more likely to stay and move in inner squares (Figure 2C–2F) in OFT.

### Spermidine and spermine alleviates oxidative stress in the brain of SAMP8

**Figure 3:**
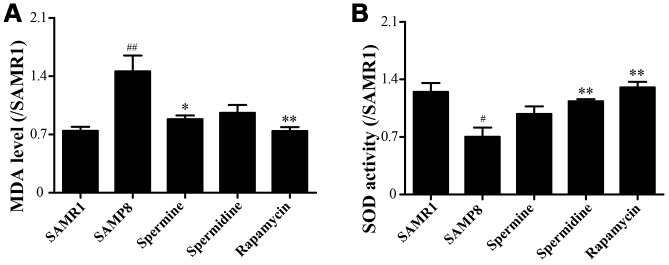
**Spermidine and spermine attenuates oxidative stress in SAMP8.** (**A**) The level of MDA in brain. (**B**) The level of ROS in the brain. Data represent mean ± SEM (*n* = 6 per group). ^#^*P* < 0.05, ^##^*P* < 0.01, ^###^*P* < 0.001 vs. SAMR1; *P < 0.05, **P < 0.01, ***P < 0.001 vs. SAMP8.

We evaluated the effect of polyamine and rapamycin on oxidative stress. Spermidine, spermine and rapamycin decreased the levels of MDA in the brain of SAMP8 mice (Figure 3A). The activity of SOD was particularly increased in the group of spermidine, spermine and rapamycin (Figure 3B). These results indicated that spermidine, spermine and rapamycin greatly ameliorate oxidative stress in SAMP8.

### Spermidine and spermine increase synaptic plasticity and neurotrophic factors in SAMP8

**Figure 4:**
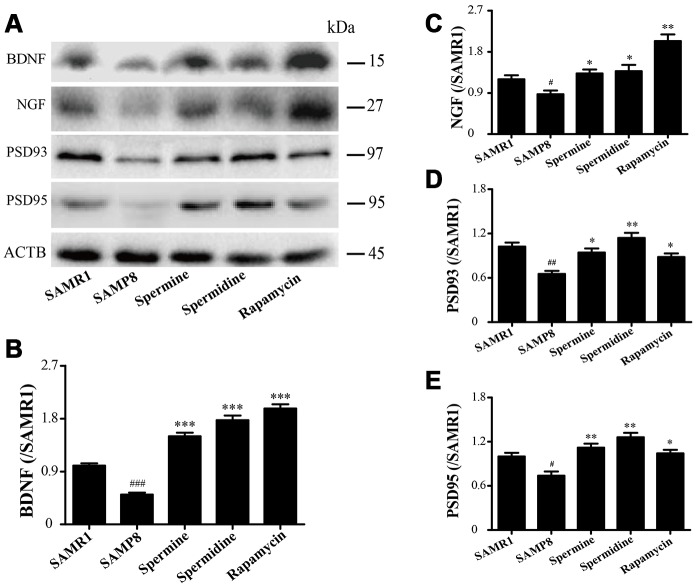
**The effect of spermidine and spermine on neurodegeneration in SAMP8.** (**A**) Western blot of NGF, PSD93, PSD95 and BDNF. (**B**) The expression of BDNF in the brain. (**C**) The expression of NGF in the brain. (**D**) The expression of PSD93 in the brain. (**E**) The expression of PSD95 in the brain. (Data represent mean ± SEM (*n* = 3 per group). ^#^*P* < 0.05, ^##^*P* < 0.01, ^###^*P* < 0.001 vs. SAMR1; *P < 0.05, **P < 0.01, ***P < 0.001 vs. SAMP8.

The expression of neurotrophic factors and synaptic proteins were detected in all groups (Figure 4). Nerve growth factor (NGF), brain-derived neurotrophic factor (BDNF), postsynaptic density-95 (PSD95) and postsynaptic density-93 (PSD93) was obviously improved in the group of spermine, spermidine and rapamycin.

### Spermidine and spermine redress mitochondrial dysfunction in SAMP8

**Figure 5:**
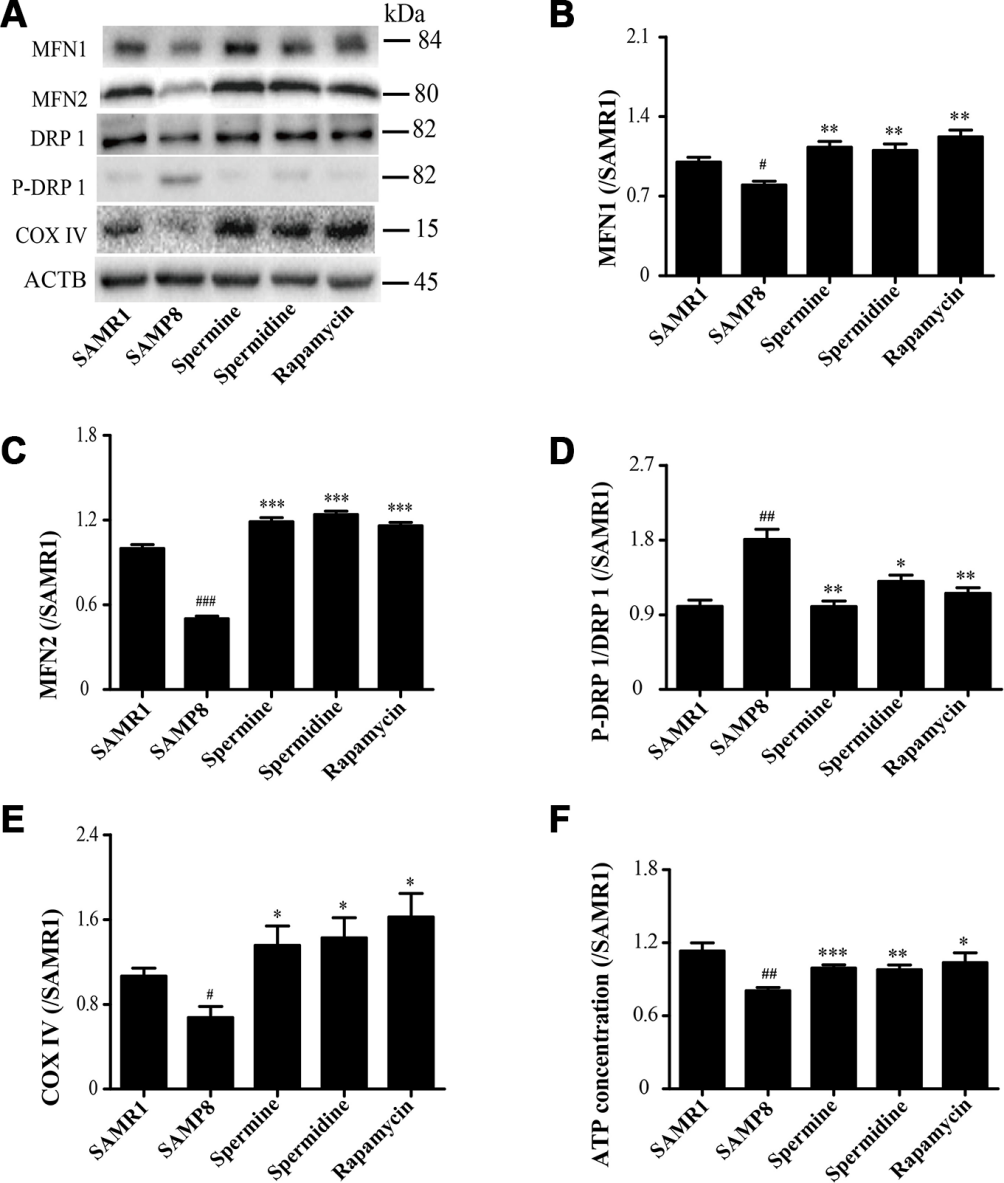
**Spermidine and spermine redress mitochondrial dysfunction in SAMP8.** (**A**) The western blot of mitochondrial protein. (**B**) The expression of MFN1 in the brain. (**C**) The expression of MFN2 in the brain. (**D**) The expression of P-DRP1 in the brain. (**E**) The expression of COX IV in the brain. (**F**) The concentration of ATP in the brain. Data represent mean ± SEM (*n* = 3 per group). ^#^*P* < 0.05, ^##^*P* < 0.01, ^###^*P* < 0.001 vs. SAMR1; *P < 0.05, **P < 0.01, ***P < 0.001 vs. SAMP8.

As shown in Figure 5, the expression of proteins related to mitochondrion, including MFN1, MFN2 and COX IV, were greatly improved in spermidine, spermine and rapamycin groups (Figure 5B, 5C, 5E). Spermidine, spermine and rapamycin obviously induced the phosphorylation of DRP 1 (Figure 5D). The concentration of ATP was lower in SAMP8 group than in spermidine, spermine and rapamycin groups (Figure 4F).

### Spermidine and spermine phosphorylate AMPK and induce autophagy in SAMP8

**Figure 6:**
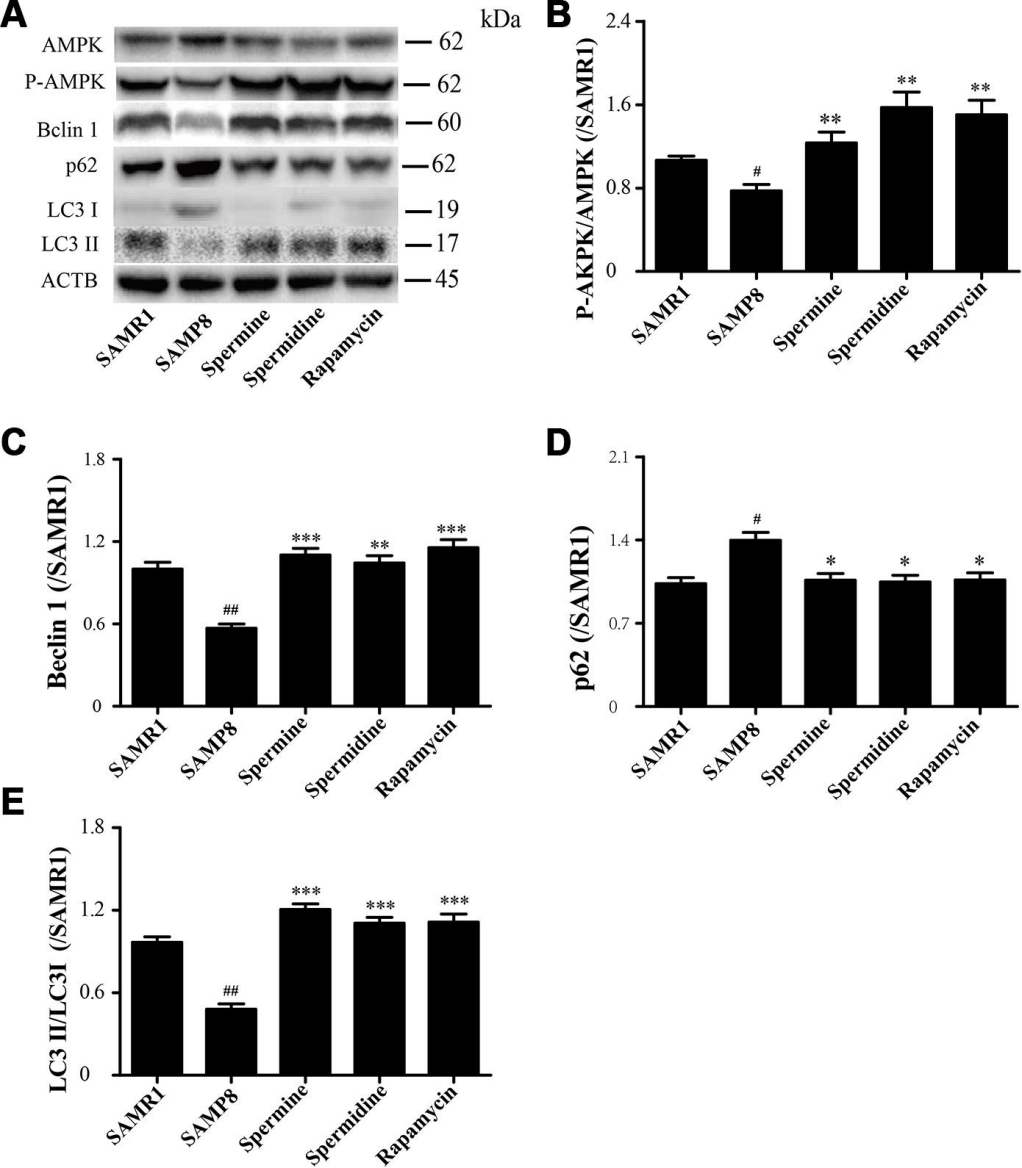
**Spermidine and spermine phosphorylate AMPK and inducing autophagy in SAMP8.** (**A**) The western blot of autophagy protein. (**B**) The expression of P-AMPK in the brain. (**C**) The expression of Beclin in the brain. (**D**) The expression of p62 in the brain. (**E**) The expression of LC3 in the brain. Data represent mean ± SEM (*n* = 3 per group). ^#^*P* < 0.05, ^##^*P* < 0.01, ^###^*P* < 0.001 vs. SAMR1; *P < 0.05, **P < 0.01, ***P < 0.001 vs. SAMP8.

The level of phosphorylated AMPK in SAMR1 group is lower than SAMP8 group. After the treatment of spermidine, spermine and rapamycin, the level of phosphorylation of AMPK was elevated (Figure 6A, 6B). The level of proteins related to autophagy including Beclin 1 and LC3II was significantly increased in spermidine, spermine and rapamycin groups (Figure 6A, 6C, 6E). The expression of p62 was lower in spermidine, spermine and rapamycin groups (Figure 6D).

### Spermidine and spermine ameliorate apoptosis in SAMP8

**Figure 7:**
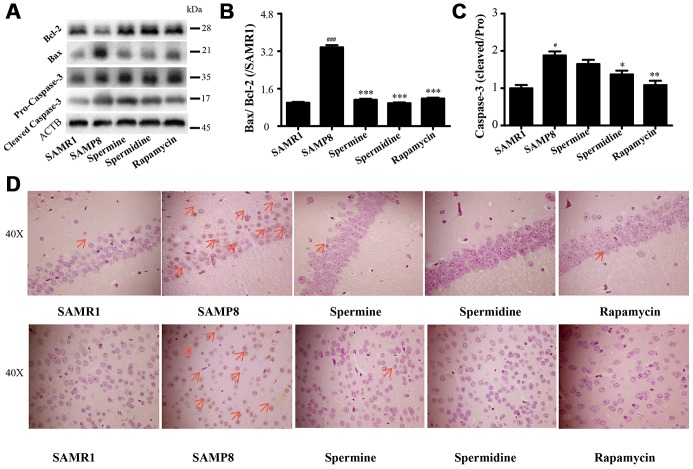
**Spermidine and spermine depress apoptosis in SAMP8.** (**A**) The western blot of apoptotic protein. (**B**) The expression of Bax and Bcl-2 in the brain. (**C**) The expression of Caspase-3 in the brain. (**D**) TUNEL staining in cortex. Scale bar: 50μm. Data represent mean ± SEM (*n* = 3 per group). ^#^*P* < 0.05, ^##^*P* < 0.01, ^###^*P* < 0.001 vs. SAMR1; *P < 0.05, **P < 0.01, ***P < 0.001 vs. SAMP8.

We detected the expression of apoptotic proteins to illuminate the effect of spermidine, spermine on neuronal apoptosis (Figure 7). The level of Bax and cleaved Caspase-3 increased and Bcl-2 decreased in SAMP8 group. Spermidine, spermine and rapamycin sharply decreased the expression of Bax (Figure 7B) and cleaved Caspase-3 (Figure 7C) and obviously increased the level of Bcl-2 (Figure 7B). Increased apoptotic cells were stained brown in SAMP8 group, compared to SAMR1 group (Figure 7B). While the TUNEL-positive cells were greatly decreased in the brain slice of spermine, spermidine and rapamycin groups in both hippocampus and cortex.

### Spermidine and spermine attenuate inflammation in SAMP8

**Figure 8:**
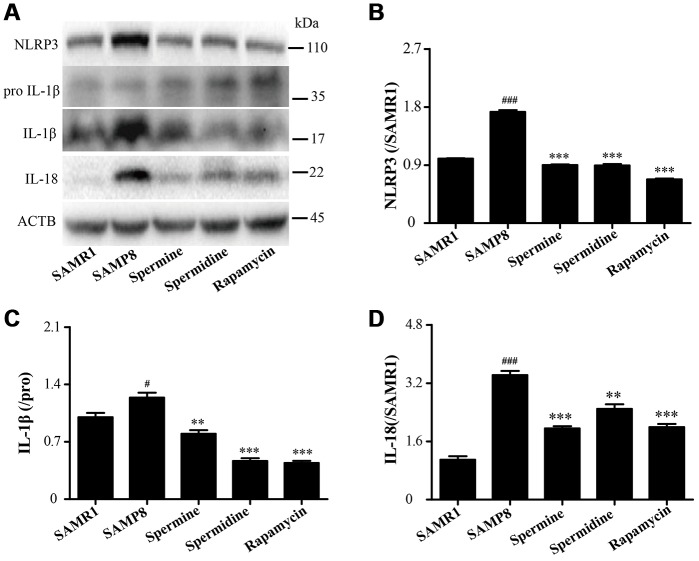
**Spermidine and spermine ameliorate inflammation in SAMP8.** (**A**) The western blot of inflammatory protein. (**B**) The expression of NLRP3 in the brain. (**C**) The expression of IL-1β in the brain. (**D**) The expression of IL-18 in the brain. Data represent mean ± SEM (*n* = 3 per group). ^#^*P* < 0.05, ^##^*P* < 0.01, ^###^*P* < 0.001 vs. SAMR1; *P < 0.05, **P < 0.01, ***P < 0.001 vs. SAMP8.

As shown in Figure 8, the level of inflammatory proteins, including NLRP3, IL-1β and IL-18 was obviously increased in SAMP8 group (Figure 8). After the treatment of spermidine, spermine and rapamycin, the expression of inflammatory proteins (NLRP3, IL-1β and IL-18) were decreased sharply. These results indicated that spermidine, spermine could depress inflammation in the brain of SAMP8 mice.

## DISCUSSION

In this study, we demonstrated that long-time administration of polyamines, spermidine and spermine, delay brain aging and improved cognitive dysfunction in SAMP8. We further explored the mechanism of polyamine spermidine and spermine in anti-brain aging.

Brain aging is characterized by cognitive dysfunction, neuronal loss and synapse loss [34–36]. At the early stage of brain aging, visual recognition memory decline is a potential marker. The ORT is designed based on the visual memory to examine the exploration of a novel object. According to the times to explore the novel object and movement paths, spermidine and spermine promoted memory retrieval. The OFT is designed to examine locomotor activity and anxious emotional based on the time spent in the inner square, distance in the inner square and the activity of lifting fore legs upwards, scratching face, and licking foot. Spermidine and spermine significantly relieve the tension of mice. PSD95 and PSD93, the major scaffolding protein at excitatory synapses and in postsynaptic densities (PSDs), are crucial for synapse maturation and plasticity [37, 38]. The expression of PSD93 and PSD95 is greatly improved after the treatment of spermidine, spermine and rapamycin, in line with the results of ORT and open field test. The expression of brain-derived neurotrophic factor (BDNF), related to synaptic plasticity, neurogenesis, learning memory and cognition [39–41], is reported to decline in neurons [42, 43]. Nerve growth factor (NGF) promotes survival and maintains the phenotype of neurons in the central nervous system (CNS) [44, 45]. In our study, the level of NGF and BDNF increased highly in the mice treated with spermidine and spermine.

Oxidative stress, imbalance in aerobic metabolism, induces reactive oxygen species (ROS) which lead to neuronal apoptosis in neurodegenerative disease [46–48]. Mitochondrial damage can also induce oxidative phosphorylation and stimulate cell death [49]. Superoxide dismutase (SOD) provides a primary defense against oxidation stress by scavenging free radicals. Malondialdehyde (MDA), an index of lipid per oxidation, indicates the overproduction of ROS [50]. In this study, spermidine, spermine and rapamycin robustly decreased MDA levels. The activity of SOD was also obviously improved in spermidine, spermine and rapamycin groups, when compared with SAMP8. All these results indicated that spermidine, spermine and rapamycin suppressed oxidative stress in SAMP8 mice.

AMP-activated kinase (AMPK), an intracellular energy sensor, promotes autophagy through the phosphorylation of AMPK [51–53]. In the brain of spermidine, spermine and rapamycin, the expression of P-AMPK is improved, compared to SAMP8. Polyamine (putrescine, spermidine and spermine; Figure 1) have been reported to decrease with age in the brain of rats and humans [54, 55]. Here we show that the administration of exogenous spermidine and spermine meliorate the cognitive dysfunction. It is reported that spermidine induced autophagy to protect age-induced memory impairment [16] and attenuated brain aging [17]. Spermine has been proved to ameliorate age-induced neurodegeneration in an autophagy-dependent manner [56]. Autophagy is a self-eating mechanism and eliminates the aggregated protein and dysfunction organelles in autolysosomes [57]. Autophagy is a multistep process that includes initiation, membrane nucleation and phagophore formation, phagophore expansion, fusion with the lysosome and degradation. LC3 family proteins are attached to autophagosomal membranes, where they participate in cargo recognition and recruitment of the phagophore by interacting with various autophagy receptors. Prominent examples of autophagy receptors are p62 (also known as SQSTM1), which recognize ubiquitinated proteins or organelles targeted for degradation. Beclin 1, the core complexes controlling autophagosomes maturation, promotes autophagy [58–60]. In our study, we can saw the expression of autophagy-related proteins, including Beclin 1 and LC 3 II, decline in SAMP8 group. Spermidine and spermine, especially rapamycin, increased Beclin 1 and LC 3 II and decreased p62 in mice brain. Based on these results, spermidine and spermine phosphorylated AMPK and then induced autophagy in SAMP8 mice.

A very important function of the mitochondrial in the cell is to produce ATP, the energy used for a variety of metabolic reactions. Cells transport damage in their nuclear and mitochondrial genomes to organelles, then the quality control mechanisms determine the consequences of the cell and the organism during aging [30]. Mitochondrial dysfunction and impaired autophagy are the hallmarks of aging [61]. Autophagy and mitochondrial biogenesis are crucial to the quality and balance of mitochondria. Mitochondrial fusion is related to various proteins, including mitofusin-1 (MFN1) and mitofusin-2 (MFN2). Mitochondrial fission is connected with dynamin-related protein 1 (DRP1) [62, 63]. Cytochrome c oxidase IV (COX IV), deficient in aging [64], is an important mitochondrial marker enzyme that is positively correlated with respiration [65]. The expression of mitochondrial fusion (MFN1 and MFN2) apparently increases in spermidine, spermine and rapamycin groups, but was accompanied with lower phosphorylation of mitochondrial fission protein DRP 1. Mitochondria maintained balance and quality through mitochondrial fission and fusion in the brain of polyamine-treating group. The ATP concentration increased in spermidine, spermine and rapamycin groups. These results demonstrated that spermidine and spermine ameliorated mitochondrial dysfunction to produce the energy for cell survival.

NLRP3, an inflammasomes receptor, can interact with Beclin 1 directly and undergo polyubiquitination which was recognized by the UBA domain of p62 protein. Subsequently, p62 targeted the NLRP3 inflammasome to the LC3-mediated autophagy [66]. NLRP3 regulates its downstream target caspase-1 and subsequent maturation of interleukin-1β (IL-1β) and IL-18 [67, 68]. Beclin 1, a coiled-coil protein, also interacts with multiple apoptotic proteins such as Bcl-2 (an anti-apoptosis protein) [60]. In this experiment, the expression of proinflammatory factor (NLRP3, IL-1β, IL-18 and caspase-1) and pro-apoptosis protein (Bax and caspase-3) and TUNEL-positive cells were sharply down-regulated in polyamine and rapamycin groups. We demonstrated that polyamine induced autophagy to attenuate inflammation and apoptosis in the aging brain.

In this study, we illustrated that polyamine could ameliorate brain aging, which might be related to autophagy and mitochondrial dysfunction. The further mechanism still needs to be studied.

## MATERIALS AND METHODS

### Mice

Three-month-old male senescence-accelerated mouse prone 8 (SAMP8) and senescence-accelerated mouse resistant 1 (SAMR1) mice were purchased from the Peking University Health Science Center. The mice were housed in a specific pathogen-free animal room. The procedures applied in the study were carried out according to the Guiding Principles for the Care and Use of Laboratory Animals that were adopted and promulgated by the United States National Institutes of Health.

### Drug treatments

Polyamine spermidine (2 mmol/L, Aladdin) and spermine (2 mmol/L, Sigma) were diluted in drinking water according to previous experiment [16]. Mice were respectively treated with saline, spermidine, spermine and rapamycin (0.78mg/kg/d, Sigma, intragastric administration) for 8 weeks.

### Object recognition test (ORT)

The mice were placed individually in the testing arena (40 cm in length, 40 cm in width, 40 cm in height), fora period of 5 minutes with no objects present. Two identical objects were placed in the central part of the chamber, equally distant from the perimeter to be tested on the next day. Interactions with both objects were recorded for 5 minutes with a digital video camera mounted overhead. The mice were placed in the chamber again with two objects (a familiar object from the preceding test and a novel object) to test memory retention on the third day. Interactions with both objects were recorded for 5 minutes. During the testing, the condition including the spatial location for the objects remained the same. After each test, the arena was cleaned with a solution of 70 % ethanol to avoid olfactory cues. Discrimination index (exploration of novel object/total exploration time), novel percent time and percent alterations were measured in this experiment.

### Open field test (OFT)

The open field test apparatus is constructed of white wooden square, which is divided into 25squares (16 peripheral and 9 central). The mice were singly placed in the center of a square arena (Med Associates Inc., St. Albans, USA, 100 cm × 100 cm) to move freely for 5 minutes. Animal behavior was videotaped by a video camera mounted overhead. After each test, the arena was cleaned with a solution of 70% ethanol.

### TUNEL staining

Brain paraffin sections were washed in xylene and rehydrated through a graded series of ethanol and double-distilled water. Then, the staining was performed according to the specification of TUNEL. Then slides were dehydrated through 70%, 95% and 100% alcohol, cleared in xylene. Images were analyzed by using a light microscope and LEICA QWin Plus (Leica Microsystems, Wetzlar, Germany).

### Western blot analysis

The brain tissues were homogenized and lysed in sample buffer (0.5 M Tris/HCl pH 6.8, 50% glycerol, 10% sodium dodecyl sulphate (SDS), 1: 100 inhibitor proteases and phosphatases cocktail) on ice. We centrifuged the lysate and then boiled at 100 °C with loading buffer. The lysate (30μg protein) was fractionated by SDS-polyacrylamide gel electrophoresis (PAGE) and then transferred onto polyvinylidene fluoride sheets (PVDF) membranes. After being blocked with 5 %Bull Serum Albumin (BSA) dissolved in TBST for one and half hour at room temperature, transferred PVDF membranes were incubated overnight at 4 °C overnight with the primary antibodies. The membrane was incubated with secondary antibody (anti-rabbit or anti-mouse) for one and half hour at room temperature. Routinely, protein load was detected by using a super enhanced chemiluminescence reagent (ECL; Applygen Technologies Inc., Beijing, China).

### Measurement of MDA, AOD, ROS and ATP

The brain tissues were centrifuged with ice-cold saline. We used the supernatant to detect the levels of ROS, SOD, MDA and ATP according to the manufacturer’s instructions. The absorbance was read using Universal Microplate Spectrophotometer (Bio-Rad, Hercules, CA, USA).

### Statistical analysis

Experimental values were given as means ± S.E.M. All statistical analysis was performed with SPSS 19.0 statistical software (IBM, Endicott, NY). Two-way analysis of variance (ANOVA) was applied to analyze differences in data for the biochemical parameters among the different groups, followed by Dunnett’s significant post-hoc test for pair-wise multiple comparisons. The level of statistical significance for all tests was *P* < 0.05.
